# Pomegranate Peel Extract and Punicalagin Are Associated with Attenuated Intestinal Barrier Dysfunction, Oxidative Stress, and Multi-Organ Injury in a Rat CLP Model

**DOI:** 10.3390/biomedicines14102223

**Published:** 2026-10-01

**Authors:** Mustafa Yeni, Hilal Ürüşan, Bülent Bayraktar, Gökşad Cemil Kotan

**Affiliations:** 1 Erzurum Regional Training and Research Hospital, 25240 Erzurum, Türkiye; 2 Department of Plant and Animal Production, Vocational School of Technical Sciences, Atatürk University, 25240 Erzurum, Türkiye; 3 Department of Physiotherapy and Rehabilitation, Faculty of Health Sciences, Bayburt University, 69000 Bayburt, Türkiye; 4 Laboratory and Veterinary Health Program, Department of Veterinary Medicine, Alaca Avni Çelik Vocational School, Hitit University, 19000 Çorum, Türkiye

**Keywords:** *Punica granatum*, pomegranate peel, punicalagin, cecal ligation and puncture, intestinal barrier, bacterial translocation, oxidative stress, immunometabolism, multi-organ dysfunction

## Abstract

**Background/Objectives**: Cecal ligation and puncture (CLP)-induced sepsis is accompanied by intestinal tight-junction disruption, circulating markers of barrier dysfunction, oxidative stress, bacterial dissemination, and multi-organ injury. This study evaluated the protective effects of chemically characterized pomegranate peel extract (PPE) and punicalagin (PUN) in a rat CLP model. **Methods**: Forty-eight female Wistar rats were randomized into six groups (*n* = 8): Sham, CLP, CLP + PPE 150 mg/kg, CLP + PPE 300 mg/kg, CLP + PUN 50 mg/kg, and non-septic PPE 300 mg/kg. Treatments were administered orally 1 h after surgery. Animals were monitored for 48 h and sampled at a predefined humane endpoint or at 48 h. **Results**: CLP reduced ileal ZO-1 and occludin abundance and increased serum diamine oxidase, D-lactate, and plasma endotoxin (all omnibus *p* < 0.001). Relative to untreated CLP, PPE 300 mg/kg increased ZO-1 and occludin by 219% and 236%, respectively, and reduced circulating markers consistent with intestinal barrier dysfunction, oxidative stress, lactate, and multi-organ injury. PPE 300 mg/kg and PUN 50 mg/kg were also associated with higher hepatic SIRT1/PGC-1α expression, lower recoverable aerobic bacterial burden, less histological injury, and higher observed 48 h survival (7/8 and 8/8 vs. 2/8 in CLP). **Conclusions**: PPE and PUN produced coordinated protective changes in this experimental CLP model. These biomarker-based and survival findings support further preclinical investigation of standardized pomegranate-derived preparations as potential adjunctive interventions; they do not establish direct intestinal permeability, mitochondrial restoration, clinical therapeutic efficacy, or a punicalagin-mediated mechanism.

## 1. Introduction

Sepsis is a life-threatening organ dysfunction caused by a dysregulated host response to infection [1]. Despite advances in early recognition, antimicrobial therapy, source control, hemodynamic support, and organ-replacement strategies, sepsis remains a major cause of global mortality and long-term disability [2,3,4]. Its pathobiology cannot be reduced to an isolated cytokine surge. Pathogen-associated and damage-associated molecular patterns simultaneously activate innate immunity, complement, coagulation, endothelial signaling, and neuroendocrine stress responses, while counter-regulatory immunosuppression may develop in parallel [4,5,6,7]. The clinical phenotype therefore reflects a dynamic interaction among inflammation, immune exhaustion, microcirculatory failure, mitochondrial dysfunction, and altered substrate utilization rather than a uniformly hyperinflammatory state.

The intestine is both an early target and a biological amplifier of systemic injury during sepsis. Splanchnic hypoperfusion, oxidative stress, inflammatory mediators, epithelial apoptosis, and altered junctional trafficking can disrupt mucosal compartmentalization and permit luminal antigens, endotoxin, and viable microorganisms to reach extra-intestinal sites [8,9,10]. Zonula occludens-1 (ZO-1) links transmembrane tight-junction proteins to the actin cytoskeleton, whereas occludin contributes to junctional stability. Circulating diamine oxidase (DAO) and D-lactate are supportive markers of enterocyte injury and barrier dysfunction, while plasma endotoxin reflects systemic exposure to Gram-negative bacterial products [8,9,10]. These measures provide convergent, but indirect, evidence and should not be interpreted as a direct permeability test.

Loss of intestinal compartmentalization may amplify a gut–vascular–organ axis. Endotoxin and disseminated bacteria can activate endothelial adhesion pathways, promote glycocalyx shedding, disturb capillary flow, and intensify pulmonary, hepatic, renal, cardiac, and neurovascular injury [7,8,9,10,11]. Syndecan-1 is released during glycocalyx degradation, E-selectin reflects endothelial activation, KIM-1 indicates renal tubular injury, and SP-D and sRAGE provide information on pulmonary epithelial disturbance [11]. Their concurrent assessment with quantitative bacterial burden and organ histology permits a multidomain evaluation of septic injury, while remaining associative.

Oxidative stress and altered metabolic signaling contribute to septic organ dysfunction. Excess reactive oxygen and nitrogen species can damage lipids, proteins, mitochondrial DNA, and respiratory-chain components, while reduced SOD, CAT, and circulating antioxidant capacity diminish tissue resilience [12,13,14]. SIRT1 and PGC-1α participate in mitochondrial biogenesis and metabolic adaptation; however, their protein abundance alone does not measure mitochondrial respiration, ATP production, membrane potential, or biogenesis. Accordingly, the present study treats SIRT1/PGC-1α as candidate signaling correlates and does not infer restored mitochondrial function from these measurements.

Pomegranate (*Punica granatum* L.) peel is a concentrated source of polyphenols, particularly the hydrolysable ellagitannins alpha- and beta-punicalagin, together with punicalin, ellagic acid, gallic acid, and flavonoids [15,16]. Punicalagin is a high-molecular-weight, water-soluble ellagitannin that occurs as alpha- and beta-anomers; its glucose core is esterified with gallagyl and hexahydroxydiphenoyl groups. This structure explains both its strong protein-binding capacity and its susceptibility to hydrolysis in biological environments. These constituents have been associated with antioxidant, anti-inflammatory, antimicrobial, and barrier-protective effects in experimental systems [17,18].

In the gastrointestinal tract, punicalagin undergoes hydrolysis to smaller ellagitannin-derived products, including ellagic acid; intestinal microbiota can then convert ellagic acid into urolithins. Because these metabolites differ in absorption, distribution, and biological activity, the effects of orally administered PPE or punicalagin may reflect both the parent compound and its downstream metabolites [19,20]. This biotransformation is relevant when interpreting systemic effects after oral dosing.

Nevertheless, the pomegranate–sepsis literature is not uniformly favorable. Makled et al. reported attenuation of CLP-induced hepatic oxidative stress and inflammation through TLR4/NF-κB pathway modulation [21]; Trovão et al. observed prolonged survival with *P. granatum* peel extract in lethal murine sepsis [22]; and a recent experimental study found that punicalagin enhanced the activity of meropenem in polymicrobial sepsis [23]. Ülker et al. likewise documented lower blood-culture positivity and histopathological injury after *P. granatum* peel powder in septic rats [24]. In contrast, Tavasoli et al. reported increased peritoneal bacterial burden and mortality under different exposure conditions [25], and prolonged high-dose pretreatment was associated with suppression of neutrophil myeloperoxidase responses [26]. These differences indicate that plant material, extraction chemistry, dose, treatment timing, CLP severity, and the balance between inflammation control and antimicrobial host defense can determine whether an antioxidant intervention is beneficial or detrimental.

Safety should not be inferred from antioxidant activity alone. Tannin-rich preparations may show dose-, matrix-, and exposure-dependent effects, including protein binding, altered nutrient availability, and modulation of antimicrobial immune functions. In the present study, the non-septic PPE 300 mg/kg group showed no detected adverse change among the measured endpoints during the 48 h observation period. This limited acute observation supports short-term tolerability only under the tested conditions; it does not establish general, chronic, reproductive, hepatic, or renal safety.

An important knowledge gap therefore remains. Previous studies have reported both beneficial and adverse effects of pomegranate-derived preparations in experimental sepsis [21,22,23,24,25,26], but few have integrated chemically characterized PPE and purified PUN across intestinal tight-junction abundance, circulating markers of barrier dysfunction, redox status, immunometabolic signaling, recoverable bacterial burden, multi-organ histology, and short-term survival within a dose-resolved design. The novelty of the present work lies in this integrated comparison, not in claiming that pomegranate-derived preparations have never been studied in sepsis.

Accordingly, this study evaluated post-CLP treatment with PPE at 150 and 300 mg/kg and PUN at 50 mg/kg in female Wistar rats. The prespecified objective was to examine whether these interventions were associated with coordinated changes across five domains: (i) tight-junction protein abundance and circulating markers consistent with intestinal barrier dysfunction; (ii) redox homeostasis; (iii) SIRT1/PGC-1α-associated and immunometabolic signaling; (iv) biochemical and histological multi-organ injury; and (v) recoverable aerobic bacterial burden and short-term survival. Because the PPE and PUN regimens were not equimolar or exposure-matched, the design cannot determine whether punicalagin quantitatively accounts for PPE activity. NLRP3/pyroptosis was not tested and is considered only a future mechanistic hypothesis.

## 2. Materials and Methods

### 2.1. Animals, Ethical Approval, and Experimental Groups

Forty-eight adult female Wistar albino rats (initial body weight: 180–200 g) were housed under standard environmental conditions (temperature: 21–24 °C; relative humidity: 40–45%; 12 h light/12 h dark cycle) with ad libitum access to standard rodent diet and drinking water. The experimental animals were obtained from the Etlik Veterinary Control Central Research Institute, Republic of Türkiye Ministry of Agriculture and Forestry (Ankara, Türkiye). All experimental procedures were conducted in strict accordance with national animal welfare regulations and ARRIVE 2.0 guidelines [27], following approval by the Local Ethics Committee for Animal Experiments of Ankara Training and Research Hospital, University of Health Sciences, Türkiye (Meeting No: 0087; Decision No: 821; 16 January 2025).

Following acclimatization, rats were randomized based on baseline body weight into six experimental groups (*n* = 8 per group):

1. Sham group: Underwent laparotomy and cecal mobilization without ligation or puncture, receiving vehicle only.
2. CLP group: Sepsis was induced by cecal ligation and double puncture without therapeutic treatment; this group served as the untreated disease control.
3. CLP + PPE 150 group: Septic rats treated with a single oral dose of pomegranate peel extract (150 mg/kg).
4. CLP + PPE 300 group: Septic rats treated with a single oral dose of pomegranate peel extract (300 mg/kg).
5. CLP + PUN 50 group: Septic rats treated with a single oral dose of purified punicalagin (50 mg/kg) as the active constituent comparator.
6. PPE per se 300 group: Non-septic rats receiving 300 mg/kg pomegranate peel extract orally to establish baseline physiological safety and rule out compound toxicity.

The study included eight female rats in each group. The submitted materials do not document a prospective power calculation or a specific rationale for selecting female animals. This modest sample size limits precision, particularly for survival estimates and small between-group differences; the study was not designed to evaluate sex effects, and the absence of male animals limits generalizability because immune and sepsis responses may be sex dependent. Animals were randomly allocated to the six groups, as reported in the original protocol. Weight-based stratification and complete individual-animal outcome accounting require confirmation from the allocation and study records.

**Figure 1:**
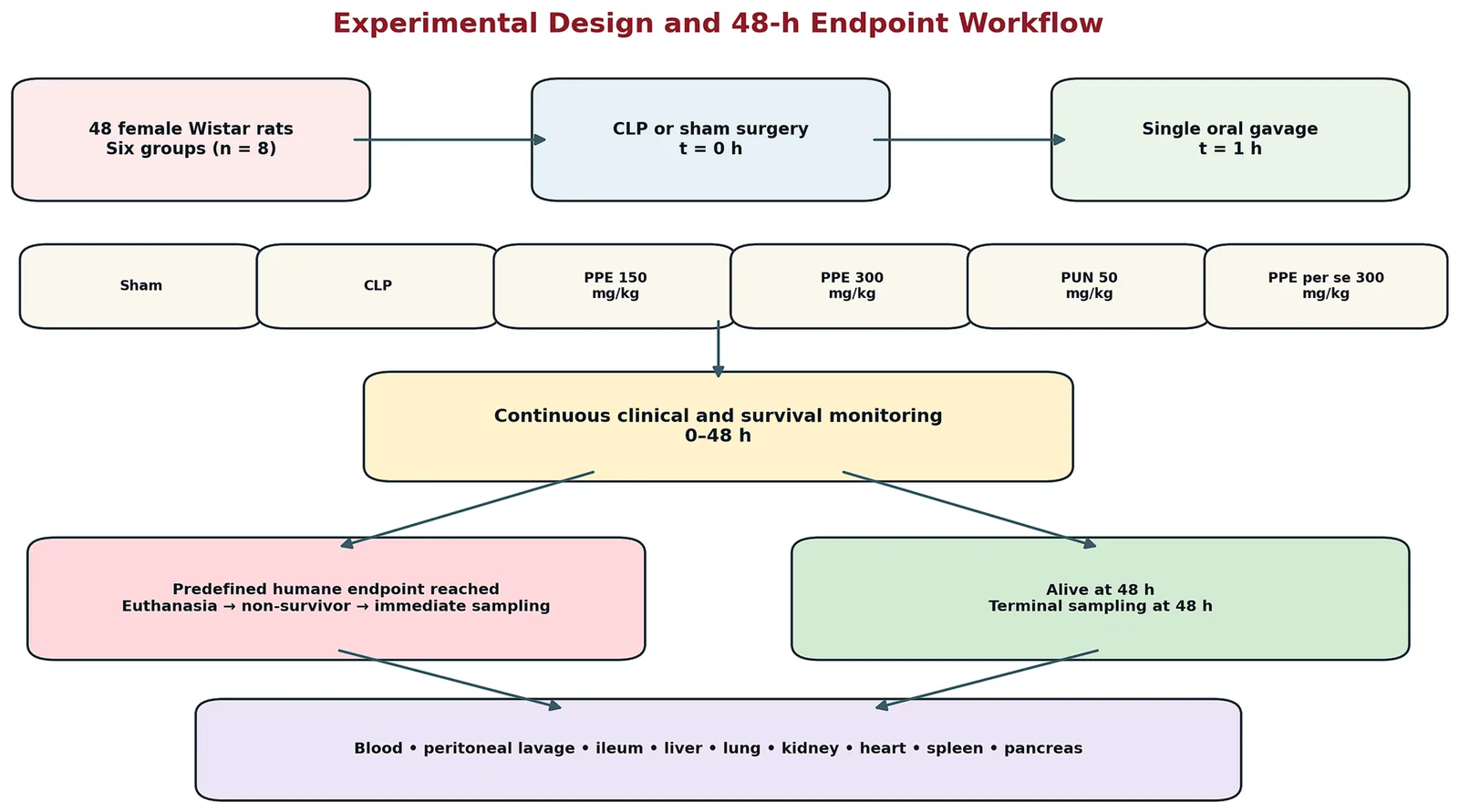
Experimental design and study workflow. Forty-eight adult female Wistar rats were randomized into six groups (*n* = 8 per group). CLP or sham surgery was performed at t = 0 h, and PPE or PUN was administered by single oral gavage at t = 1 h. Animals were monitored continuously for up to 48 h. Rats reaching a predefined humane endpoint were euthanized, counted as non-survivors, and sampled immediately; animals completing follow-up were sampled at 48 h. PPE, pomegranate peel extract; PUN, punicalagin; CLP, cecal ligation and puncture.

All treatments were administered by oral gavage 1 h after CLP or sham surgery. Animals were monitored continuously for clinical status and survival for up to 48 h. Rats reaching a predefined humane endpoint were euthanized, recorded as non-survivors, and sampled immediately; survivors were sampled at 48 h. Thus, terminal biomarker, culture, and histology measurements were obtained at different post-CLP times according to clinical course and may be confounded by sampling time. The institutional ethics committee providing protocol oversight is independent of author affiliation; the approving body and authorization identifiers are reported exactly as issued. The study workflow is shown in Figure 1.

### 2.2. Induction of Sepsis (Cecal Ligation and Puncture Model)

Polymicrobial sepsis was induced using the cecal ligation and puncture (CLP) model as described previously [28]. Briefly, rats were anesthetized via intraperitoneal injection of ketamine (50 mg/kg) and xylazine (10 mg/kg). Following standard aseptic preparation, a 3 cm midline laparotomy was performed to exteriorize the cecum. The cecum was ligated with 4-0 silk distal to the ileocecal junction, ensuring that ileocecal patency and intestinal transit remained unobstructed. Sepsis was initiated by performing a double puncture on the antimesenteric border of the ligated cecal segment using an 18-gauge needle, followed by the gentle extrusion of a small droplet of fecal content to verify patency and initiate polymicrobial peritonitis. The cecum was subsequently returned to the peritoneal cavity, and the abdominal wall was closed in two anatomical layers with surgical sutures. Sham-operated control animals underwent an identical laparotomy and cecal exteriorization without ligation or puncture.

### 2.3. Pomegranate Preparations and HPLC-DAD Standardization

Pomegranate peel extract (PPE) was prepared from mature *Punica granatum* L. fruits. The available study records do not contain a voucher specimen number, cultivar, collection coordinates, harvest date, or separate taxonomic-authentication record; botanical provenance therefore cannot be independently verified from the submitted materials. Peels were shade-dried at room temperature (22–25 degrees C) for 7 days, ground, and extracted with 70% ethanol (1:10, *w*/*v*) by maceration for 48 h. The extract was filtered, concentrated under reduced pressure at 40 degrees C, and lyophilized. The extraction yield was 28.4% (*w*/*w*). The extract was characterized by HPLC-DAD (C18 column; water/0.1% formic acid and acetonitrile gradient; detection at 260 nm). The chromatographic source files and external-standard validation records were not included in the available materials and require confirmation from the original analytical archive. The reported profile was total punicalagin 348.6 +/− 12.4 mg/g extract (alpha-punicalagin 162.3 +/− 6.8 mg/g; beta-punicalagin 186.3 +/− 7.1 mg/g), ellagic acid 21.7 +/− 1.9 mg/g, and gallic acid 8.4 +/− 0.7 mg/g. A batch-specific certificate of analysis was not available. PPE was freshly dissolved in distilled water before administration.

### 2.4. Treatment Protocol, Experimental Timeline, and Analytical Sampling

Following CLP or sham surgery (t = 0 h), animals were returned to their home cages for post-anesthetic recovery. PPE (150 or 300 mg/kg) or PUN (50 mg/kg) was administered once by oral gavage at t = 1 h. Non-septic baseline-safety animals received PPE 300 mg/kg, whereas Sham and untreated CLP groups received an equal volume of saline vehicle. No standard-of-care antibiotic comparator was included; consequently, the study evaluates the isolated preclinical effects of PPE/PUN rather than benefit beyond antimicrobial therapy. Animals were monitored for 48 h for survival, clinical sepsis score, behavioral impairment, rat grimace score, and stool consistency. The available record does not support adding unverified details regarding postoperative fluids, temperature support, or analgesic regimens beyond the approved welfare protocol; this reporting limitation is acknowledged.

Animals were monitored for up to 48 h using predefined clinical and humane-endpoint criteria. Rats showing irreversible moribundity were euthanized and recorded as non-survivors at the humane endpoint; survivors underwent terminal procedures at 48 h. Blood, peritoneal lavage, ileum, liver, lung, kidney, heart, spleen, and pancreas were then collected. This welfare-compliant design avoided prolonged distress and post-mortem autolysis but generated outcome-dependent sampling times. Accordingly, cross-sectional terminal measurements compare peak-severity samples from animals reaching humane endpoints with 48 h samples from survivors and cannot isolate treatment effects from time-since-CLP effects.

### 2.5. Quantification of Intestinal Barrier Integrity and Endotoxemia

Distal ileal segments were harvested, thoroughly flushed with ice-cold phosphate-buffered saline (PBS, pH 7.4), weighed, and homogenized in ice-cold lysis buffer (1:10 *w*/*v*) containing a comprehensive protease inhibitor cocktail. Homogenates were centrifuged at 10,000× *g* for 15 min at 4 °C, and supernatants were collected for total protein normalization using the Bradford protein assay. Tight-junction abundance markers, Zonula Occludens-1 (ZO-1; Cat. No. E-EL-R0968; Elabscience Biotechnology Inc., Houston, TX, USA) and Occludin (Cat. No. E-EL-R0691; Elabscience Biotechnology Inc.), were measured in protein-normalized ileal homogenates using rat-specific sandwich ELISA kits and expressed as µg/mg total protein. Enterocyte injury and circulating markers consistent with barrier dysfunction were assessed in serum via Rat Diamine Oxidase (DAO; Cat. No. E0374Ra; Shanghai Korain Biotech Co., Ltd., Shanghai, China; expressed as U/L) and Rat D-Lactate (D-LA; Cat. No. SG-20912; SinoGeneClon Biotech Co., Ltd., Hangzhou, China; expressed as µg/mL) ELISA kits. Circulating plasma endotoxin/lipopolysaccharide (LPS) concentrations were determined using a chromogenic Limulus Amebocyte Lysate (LAL) endpoint assay kit (Cat. No. HIT302; Hycult Biotech, Uden, The Netherlands) and reported as endotoxin units per milliliter (EU/mL).

### 2.6. Quantification of Redox Homeostasis, Metabolic Regulators, and Multi-Organ Injury Biomarkers

Serum malondialdehyde (MDA; nmol/mL), superoxide dismutase (SOD; U/mL), and catalase (CAT; kU/L) activities, together with total antioxidant status (TAS; mmol Trolox Eq/L) and total oxidant status (TOS; µmol H_2_O_2_ Eq/L), were measured using commercial colorimetric assay kits (Rel Assay Diagnostics, Gaziantep, Türkiye) on an automated spectrophotometric analyzer, after which the systemic oxidative stress index (OSI) was calculated using the standardized formula: OSI (AU) = [TOS (µmol H_2_O_2_ Eq/L)/TAS (µmol Trolox Eq/L)] × 100 = TOS/TAS × 0.1. Hepatic homogenates were prepared to quantify mitochondrial biogenesis regulators Sirtuin-1 (SIRT1; Cat. No. E1863Ra; Shanghai Korain Biotech Co., Ltd.) and Peroxisome Proliferator-Activated Receptor-γ Coactivator-1α (PGC-1α; Cat. No. SG-20541; SinoGeneClon Biotech Co., Ltd.), with values normalized to tissue protein and expressed as ng/mg protein. Circulating organ-injury, neurovascular, and metabolic biomarkers were determined using validated rat-specific sandwich ELISA kits in strict accordance with the manufacturers’ instructions: Kidney Injury Molecule-1 (KIM-1; Cat. No. E0549Ra; range: 0.05–10 ng/mL; sensitivity: 0.01 ng/mL; Shanghai Korain Biotech Co., Ltd.), Syndecan-1/SDC1 (Cat. No. E2272Ra; range: 0.1–40 ng/mL; sensitivity: 0.058 ng/mL; Shanghai Korain Biotech Co., Ltd.), Surfactant Protein-D (SP-D; Cat. No. ELK11147; range: 0.16–10 ng/mL; sensitivity: 0.065 ng/mL; ELK Biotechnology Co., Ltd., Wuhan, China), soluble Receptor for Advanced Glycation End-products (sRAGE; Cat. No. ELK2241; range: 15.63–1000 pg/mL; ELK Biotechnology Co., Ltd.), E-Selectin/CD62E (Cat. No. E-EL-R0370; range: 0.16–10 ng/mL; Elabscience Biotechnology Inc.), Cardiac Troponin I (cTnI; Cat. No. YLA0110RA; range: 0.1–30 ng/mL; sensitivity: 0.06 ng/mL; YL Biont, Shanghai, China), Protein S100-B (Cat. No. E1360Ra; range: 5–1500 pg/mL; sensitivity: 2.61 pg/mL; Shanghai Korain Biotech Co., Ltd.), Brain-Derived Neurotrophic Factor (BDNF; Cat. No. SG-20200; range: 3–100 ng/L; sensitivity: 1 ng/L; SinoGeneClon Biotech Co., Ltd.), Neuron-Specific Enolase (NSE; Cat. No. E-EL-R0058; range: 0.31–20 ng/mL; sensitivity: 0.19 ng/mL; Elabscience Biotechnology Inc.), Resistin (Cat. No. E0211Ra; range: 5–1000 pg/mL; sensitivity: 2.52 pg/mL; Shanghai Korain Biotech Co., Ltd.), Nesfatin-1 (Cat. No. E0878Ra; range: 30–9000 ng/L; sensitivity: 16.23 ng/L; Shanghai Korain Biotech Co., Ltd.), Visfatin/NAMPT (Cat. No. E0471Ra; range: 5–2000 ng/L; sensitivity: 2.49 ng/L; Shanghai Korain Biotech Co., Ltd.), Myonectin/CTRP15 (Cat. No. E1364Ra; Shanghai Korain Biotech Co., Ltd.), Irisin/FNDC5 (Cat. No. SG-20179; range: 2–90 ng/mL; sensitivity: 0.2 ng/mL; SinoGeneClon Biotech Co., Ltd.), and CTRP3 (Cat. No. E2145Ra; Shanghai Korain Biotech Co., Ltd.). Routine serum biochemical parameters (AST, ALT, BUN, Creatinine, CK-MB, glucose, and lactate) were measured on an automated clinical chemistry autoanalyzer using certified diagnostic reagent kits. Microplate optical density (OD) readings were recorded at 450 nm using a BioTek Epoch microplate spectrophotometer (BioTek Instruments, Inc., Winooski, VT, USA), and analyte concentrations were calculated from standard regression curves run in parallel.

### 2.7. Quantitative Microbiological Analysis and Bacterial Translocation

To assess the extent of polymicrobial dissemination, extra-abdominal bacterial translocation, and recoverable aerobic bacterial burden, peritoneal lavage fluid along with aseptically excised spleen and lung tissues were harvested at each animal’s terminal endpoint (predefined humane endpoint or 48 h) under strict sterile conditions. Harvested tissue samples were immediately weighed and homogenized in sterile ice-cold isotonic saline (1:10 *w*/*v*) using a sterile tissue homogenizer. Tenfold serial dilutions of both peritoneal lavage fluid and organ homogenates were prepared in sterile phosphate-buffered saline (PBS, pH 7.4) and duplicate 100 µL aliquots were inoculated onto non-selective nutrient agar, 5% sheep blood agar, and selective MacConkey agar plates for aerobic and facultative-anaerobic bacterial enumeration under aerobic incubation. All agar plates were incubated aerobically at 37 °C for 24–48 h, after which distinct colony-forming units were enumerated using a digital colony counter and normalized to express bacterial burden as log10 CFU/mL for peritoneal lavage fluid and log10 CFU/g wet weight for solid organ homogenates. The lower analytical detection limit was set at 1.00 log10 CFU per reported unit, with values falling below this threshold recorded as under the limit of quantification rather than sterilization. Presumptive phenotypic identification of representative clinical isolates, including *Staphylococcus aureus* and *Escherichia coli*, was confirmed through characteristic colony morphology, Gram staining, catalase and coagulase activity tests, and standard automated biochemical profiling panels.

### 2.8. Histopathological Processing and Multi-Organ Injury Scoring

To comprehensively evaluate sepsis-induced multi-organ structural damage and tissue integrity at the light microscopic level, liver, lung, kidney, heart, spleen, and pancreas tissues were harvested at each animal’s terminal endpoint (predefined humane endpoint or 48 h) and immediately immersed in 10% neutral buffered formalin for 48 h. Following standardized fixation, tissue samples underwent automated dehydration through an ascending ethanol series, clearing in xylene, and embedding in paraffin wax blocks. Paraffin-embedded specimens were sectioned at a uniform thickness of 4–5 µm using a rotary microtome, mounted on silane-coated glass slides, deparaffinized, rehydrated, and stained with hematoxylin and eosin (H&E) according to routine histopathological protocols. Histomorphological alterations—including parenchymal vacuolar degeneration, interstitial edema, microvascular congestion, inflammatory polymorphonuclear leukocyte infiltration, and focal necrotic areas—were systematically evaluated across all harvested organs under an Olympus BX53 light microscope equipped with a calibrated digital image acquisition system by an experienced pathologist blinded to the experimental group allocations. The reported grades categorize structural damage according to the percentage of affected tissue area: 0 (absent/minimal injury, <10% of tissue section), 1 (mild structural damage, 10–30% of tissue section), 2 (moderate tissue alterations, 30–50% of tissue section), and 3 (severe diffuse necrosis and destruction, >50% of tissue section). Representative high-resolution photomicrographs (100× magnification) were captured to construct the comprehensive multi-organ comparative histological panel across all experimental cohorts. The original manuscript reports blinded assessment by an experienced pathologist. The number of sections and microscopic fields assessed for each animal and any additional observers are not documented in the submitted materials; these details require confirmation from pathology records. The photomicrographs are illustrative.

### 2.9. Statistical Analysis

Continuous outcomes are reported as mean ± SD (*n* = 8/group). Normality and variance assumptions were assessed before one-way ANOVA with Tukey-adjusted pairwise comparisons. Ordinal outcomes were evaluated by Kruskal–Wallis tests with Dunn-adjusted comparisons. Kaplan–Meier curves and the log-rank test were used for 48 h survival. Because this exploratory study evaluated numerous correlated endpoints, table-level omnibus *p* values do not constitute biomarker-family multiplicity control; findings are interpreted as convergent exploratory evidence rather than independent mechanistic confirmations. Individual-animal measurements and event times were not supplied with the reviewed files; effect-size confidence intervals, false-discovery-rate adjustments, and time-to-event estimates cannot be checked or recomputed here without those records. Values below 1.00 log10 CFU were retained as below the analytical quantification limit. Two-sided *p* < 0.05 defined statistical significance; analyses were performed in GraphPad Prism 9.0.

## 3. Results

### 3.1. Intestinal Barrier Integrity and Systemic Endotoxemia

Sepsis induction via CLP resulted in lower tight-junction protein abundance and higher circulating markers consistent with intestinal barrier dysfunction relative to the Sham cohort (all *p* < 0.001; Table 1). Specifically, ileal tight junction structural proteins Zonula Occludens-1 (ZO-1) and occludin were markedly depleted in the untreated CLP group, exhibiting 74.6% and 76.3% reductions compared with Sham animals (3.6 ± 0.4 vs. 14.2 ± 1.1 µg/mg protein and 2.8 ± 0.3 vs. 11.8 ± 0.9 µg/mg protein, respectively). Concomitantly, circulating markers of mucosal breakdown—serum diamine oxidase (DAO), serum D-lactate, and plasma endotoxin—demonstrated 4.70-, 6.81-, and 11.75-fold increases over Sham baseline levels, peaking at 86.5 ± 7.2 U/L, 28.6 ± 2.4 µg/mL, and 0.94 ± 0.08 EU/mL, respectively.

Post-induction intervention with pomegranate peel extract (PPE) was associated with less severe barrier-related biomarker changes in a dose-dependent manner. Compared to untreated CLP animals, high-dose PPE (300 mg/kg) substantially restored ileal ZO-1 (11.5 ± 1.0 µg/mg protein, +219.4%) and occludin levels (9.4 ± 0.8 µg/mg protein, +235.7%), while significantly decreasing serum DAO (31.2 ± 2.9 U/L, −63.9%), D-lactate (8.7 ± 0.8 µg/mL, −69.6%), and systemic endotoxemia (0.21 ± 0.02 EU/mL, −77.7%; *p* < 0.05). Administration of purified punicalagin (PUN, 50 mg/kg) produced comparable and statistically robust changes in tight-junction abundance and circulating barrier-dysfunction markers, yielding significant elevations in ZO-1 (12.1 ± 0.9 µg/mg protein, +236.1%) and occludin (10.1 ± 0.9 µg/mg protein, +260.7%), with circulating endotoxin levels dropping by 80.9% (0.18 ± 0.02 EU/mL; *p* < 0.05 vs. CLP). The PPE per se (300 mg/kg) control cohort exhibited tight junction and permeability profiles statistically indistinguishable from the Sham group across all measured parameters (*p* > 0.05), showing no detected evidence of baseline mucosal toxicity.

### 3.2. Circulating Redox Balance and Sentinel Neuro-Pulmonary Injury

Polymicrobial sepsis induction triggered severe systemic oxidative stress and impaired endogenous enzymatic antioxidant defenses relative to Sham baselines (all *p* < 0.001; Table 2). Untreated CLP rats exhibited substantial increases in serum MDA (7.45 ± 0.62 nmol/mL), TOS (29.8 ± 2.5 µmol H_2_O_2_ Eq/L), and systemic OSI (4.14 ± 0.36 AU), alongside prominent depletion of SOD (9.1 ± 0.8 U/mL), CAT (11.4 ± 1.0 kU/L), and TAS (0.72 ± 0.06 mmol Trolox Eq/L). Therapeutic intervention with PPE mitigated oxidative burden in a dose-dependent fashion. High-dose PPE (300 mg/kg) decreased MDA by 64.4% (2.65 ± 0.22 nmol/mL), TOS by 60.4% (11.8 ± 1.0 µmol H_2_O_2_ Eq/L), and reduced OSI by 83.8% (0.67 ± 0.06 AU) compared to untreated CLP, concurrently elevating SOD (+100.0%), CAT (+102.6%), and TAS (+143.1%; *p* < 0.05). Administration of purified PUN (50 mg/kg) produced a concordant antioxidant profile, achieving the lowest oxidative stress index among the intervention cohorts (OSI: 0.54 ± 0.05 AU; −87.0% vs. CLP). Circulating neurovascular (S100B) and alveolar epithelial (SP-D) disruption markers decreased by 70.9% and 67.9% in the CLP + PPE 300 group, and by 74.1% and 72.1% in the CLP + PUN 50 group (*p* < 0.05 vs. CLP), accompanied by lower blood–brain barrier and alveolar–capillary barrier degradation. No significant alterations in baseline redox or sentinel tissue markers were observed in the PPE per se (300 mg/kg) control cohort (*p* > 0.05 vs. Sham).

### 3.3. Mitochondrial Biogenesis, Endothelial Glycocalyx Integrity, and Immunometabolic Profiling

CLP sepsis induced profound mitochondrial down-regulation, shedding of the endothelial glycocalyx, acute tubular injury, and systemic metabolic dysregulation (all *p* < 0.001; Table 3 and Table 4). In untreated CLP rats, hepatic SIRT1 (2.1 ± 0.2 ng/mg protein) and PGC-1α (1.4 ± 0.1 ng/mg protein) were suppressed by 75.0% and 77.4% compared with Sham levels, while circulating myonectin was depleted by 70.7% (14.2 ± 1.3 ng/mL vs. 48.5 ± 4.2 ng/mL). Conversely, vascular syndecan-1 and renal tubular KIM-1 rose 8.00-fold (16.8 ± 1.5 ng/mL) and 7.52-fold (845.6 ± 72.4 pg/mL), respectively. Treatment with PPE (300 mg/kg) restored hepatic SIRT1 (+223.8%) and PGC-1α (+250.0%), increased myonectin (+173.9%), and reduced syndecan-1 and KIM-1 levels by 69.0% and 68.6% (*p* < 0.05 vs. CLP). Active constituent comparison with PUN (50 mg/kg) yielded statistically equivalent improvements in mitochondrial biogenesis markers and organ integrity indices (Table 3).

Immunometabolic profiling revealed pronounced elevations in pro-inflammatory adipokines alongside severe depletion of metabolic protective factors in the untreated CLP group (Table 4). Serum visfatin/NAMPT (28.4 ± 2.6 ng/mL) and resistin (24.8 ± 2.2 ng/mL) increased 4.58- and 4.86-fold relative to Sham, whereas protective CTRP3 (−72.9%), irisin (−75.6%), and nesfatin-1 (−72.8%) were depleted. Oral administration of PPE (300 mg/kg) significantly suppressed visfatin (9.8 ± 0.9 ng/mL, −65.5%) and resistin (8.4 ± 0.8 ng/mL, −66.1%), while substantially augmenting CTRP3 (118.2 ± 10.5 ng/mL, +206.2%), irisin (6.9 ± 0.6 ng/mL, +228.6%), and nesfatin-1 (2.45 ± 0.22 ng/mL, +188.2%; *p* < 0.05). PUN (50 mg/kg) produced a concordant immunometabolic regulatory pattern (Table 4).

### 3.4. Hepatic, Renal, Cardiac, and Metabolic Dysfunction

Polymicrobial sepsis induced comprehensive multi-organ functional failure accompanied by severe metabolic decompensation (all *p* < 0.001; Table 5). In the untreated CLP cohort, hepatocellular enzymes AST (312.4 ± 26.8 U/L) and ALT (162.5 ± 14.2 U/L), renal retention markers Creatinine (1.85 ± 0.16 mg/dL) and BUN (71.2 ± 6.4 mg/dL), and myocardial necrosis indicators cTnI (0.74 ± 0.07 ng/mL) and CK-MB (524.6 ± 42.5 U/L) rose 4.31-, 4.67-, 4.87-, 4.62-, 24.67-, and 3.88-fold relative to Sham baselines. Concurrently, septic rats exhibited profound hypoglycemia (54.8 ± 4.9 mg/dL vs. 118.4 ± 9.2 mg/dL in Sham) and hyperlactatemia (6.42 ± 0.58 mmol/L vs. 1.35 ± 0.12 mmol/L in Sham). Post-treatment with PPE (300 mg/kg) reversed organ injury markers: AST (−65.3%), ALT (−70.3%), Creatinine (−68.6%), BUN (−65.4%), cTnI (−85.1%), CK-MB (−63.3%), and serum lactate (−68.1%), while elevating serum glucose (+90.7%; *p* < 0.05 vs. CLP). PUN (50 mg/kg) provided equivalent cytoprotective efficacy across all functional organ domains (Table 5).

### 3.5. Neurovascular, Pulmonary Epithelial, and Endothelial Activation Markers

Sepsis induced marked concurrent increases in circulating neurovascular damage markers (S100B, NSE), pulmonary epithelial disruption markers (SP-D, sRAGE), and endothelial adhesion molecules (E-selectin), alongside depletion of neuroprotective BDNF (all *p* < 0.001; Table 6). In the untreated CLP group, S100B (268.4 ± 24.1 pg/mL), NSE (21.40 ± 1.95 ng/mL), SP-D (54.2 ± 4.8 ng/mL), sRAGE (382.5 ± 32.4 pg/mL), and E-selectin (16.95 ± 1.45 ng/mL) were markedly elevated, whereas BDNF was reduced by 70.1% (98.4 ± 9.2 pg/mL vs. 328.6 ± 26.4 pg/mL in Sham). Treatment with PPE (300 mg/kg) significantly attenuated S100B (−70.9%), NSE (−72.9%), SP-D (−67.9%), sRAGE (−68.5%), and E-selectin (−71.1%), while elevating BDNF (+189.1%; *p* < 0.05 vs. CLP). PUN (50 mg/kg) produced a matching response profile, restoring circulating BDNF levels to a range statistically indistinguishable from the Sham cohort (296.8 ± 25.8 pg/mL; *p* > 0.05).

### 3.6. Quantitative Bacterial Translocation Across Anatomical Compartments

Untreated CLP resulted in heavy bacterial dissemination within the peritoneal cavity (5.84 ± 0.42 log10 CFU/mL) and substantial systemic extra-abdominal translocation to solid reticuloendothelial and pulmonary filtration organs (spleen: 4.62 ± 0.38 log10 CFU/g; lung: 3.95 ± 0.31 log10 CFU/g; all *p* < 0.001 vs. Sham; Table 7). Treatment with PPE demonstrated dose-dependent bacterial clearance: PPE (300 mg/kg) suppressed peritoneal counts to 1.85 ± 0.16 log10 CFU/mL, lowered splenic burden to 1.20 ± 0.12 log10 CFU/g, and cleared pulmonary dissemination below the analytical quantification threshold (<1.00 log10 CFU/g; *p* < 0.05 vs. CLP; Table 7). Administration of purified PUN (50 mg/kg) achieved complete extra-abdominal bacterial clearance (<1.00 log10 CFU/g in both spleen and lungs; Table 7). Animals in the Sham and PPE per se (300 mg/kg) groups exhibited bacterial counts below the analytical limit of quantification across all anatomical compartments (Table 7).

### 3.7. In Vivo Clinical Sepsis Progression, Behavioral Impairment, Pain Scoring, and Stool Dynamics

Surgical induction of polymicrobial sepsis triggered pronounced deterioration in overall clinical appearance, severe behavioral suppression, acute facial pain responses, and enteric motility disturbances over the 48 h observational period (Table 8). Untreated CLP rats exhibited severe lethargy and prostration (behavioral score: 4), marked orbital tightening and whisker distortion (grimace pain score: 3), and profuse watery diarrhea (Table 8). The composite clinical sepsis score in the untreated CLP group reached 3.8 ± 0.4 (*p* < 0.001 vs. Sham) (Table 8).

Post-surgical oral administration of PPE attenuated clinical disease severity in a dose-dependent manner (Table 8). High-dose PPE (300 mg/kg) and purified PUN (50 mg/kg) suppressed composite clinical scores to 0.8 ± 0.2 and 0.6 ± 0.2, respectively (*p* < 0.001 vs. CLP), while restoring alertness, resolving abnormal orbital grimace manifestations, and maintaining physiological, formed stool consistency (Table 8). These multidimensional clinical improvements paralleled the enhanced 48 h cumulative survival rates observed in the PPE 300 mg/kg (87.5%) and PUN 50 mg/kg (100%) cohorts (*p* < 0.01; Table 8). Animals receiving PPE per se (300 mg/kg) exhibited normal behavioral, clinical, and fecal profiles indistinguishable from the Sham group (Table 8).

### 3.8. Multi-Organ Histopathological Evaluation

**Figure 2:**
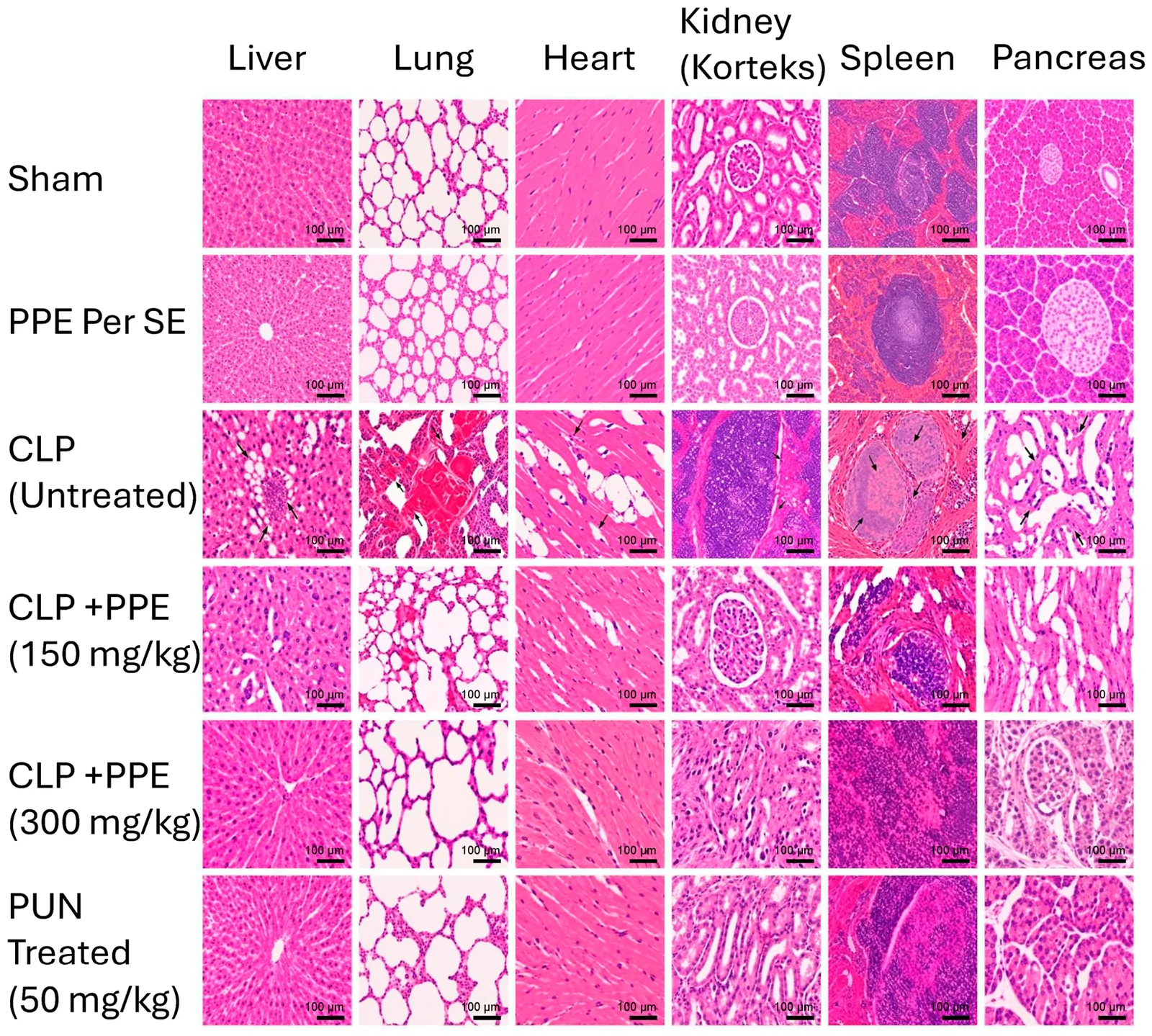
Representative H&E-stained sections of liver, lung, heart, kidney, spleen, and pancreas from Sham, PPE Per Se, CLP Untreated, CLP + PPE 150 mg/kg, CLP + PPE 300 mg/kg, and PUN-treated 50 mg/kg groups. In the displayed fields, untreated CLP tissues show inflammatory and structural abnormalities, whereas the treatment-group fields show less prominent lesions. These representative images are descriptive and do not independently demonstrate a dose–response relationship; quantitative inference should rely on the blinded histopathology scores. Original magnification ×100; scale bar = 100 micrometers. Because the displayed renal fields may not represent the same anatomical compartment across all groups, direct visual comparison of those panels should be interpreted cautiously. The number of sections and fields used for grading should be verified against the original pathology records. Black arrows indicate histopathological alterations, including inflammatory cell infiltration, tissue vacuolization, and structural degeneration in the untreated CLP group. Hematoxylin and eosin (H&E) staining; scale bars = 100 µm.

Light microscopic examination of H&E-stained tissue sections revealed severe histomorphological injury across major target organs in the untreated CLP group (Table 9; Figure 2). CLP induced extensive hepatocellular hydropic degeneration and focal necrosis (grade +++), intra-alveolar hemorrhage, neutrophil infiltration, and interalveolar septal thickening in the lungs (grade +++), severe renal tubular epithelial vacuolation, luminal cast formation, and loss of brush borders (grade +++), and marked splenic lymphoid follicle expansion with white pulp hyperplasia (grade +++), alongside moderate myocardial myofibrillar degeneration (grade ++) and pancreatic interstitial edema (grade ++).

Administration of PPE attenuated these histomorphological alterations in a dose-dependent manner: PPE (150 mg/kg) decreased injury scores across all organs by one grade, whereas high-dose PPE (300 mg/kg) was associated with milder parenchymal injury, limiting structural damage to mild (grade +) or absent-to-mild (grade −/+) levels. PUN (50 mg/kg) was associated with largely preserved organ architecture, completely resolving pancreatic injury (grade −) and yielding minimal focal changes in cardiac and hepatic tissues (grade −/+; Table 9). Tissues from Sham and PPE per se (300 mg/kg) cohorts exhibited normal cellular morphology without structural alterations.

## 4. Discussion

This study provides an integrated, dose-resolved comparison of PPE and PUN across barrier-related, redox, immunometabolic, microbial, organ-injury, histological, and survival domains in experimental CLP sepsis. The principal interpretation is not that a single pathway was proven, but that several independently measured domains changed in a concordant direction after treatment. This coherence supports biological plausibility while remaining subject to the small sample, non-equimolar exposure, outcome-dependent terminal sampling, absence of an antibiotic arm, and biomarker-based nature of several mechanistic claims.

Intestinal barrier function depends on epithelial viability, junctional localization, perfusion, and immune–microbial homeostasis [8,9,10]. The concordant changes in ileal ZO-1/occludin abundance and circulating DAO, D-lactate, and endotoxin are consistent with less severe barrier dysfunction after PPE/PUN. They do not, however, directly demonstrate permeability because FITC-dextran, Evans blue, transepithelial flux, or junctional immunolocalization was not performed. The findings are therefore described as preservation of tight-junction protein abundance accompanied by lower circulating markers consistent with barrier dysfunction.

These results agree directionally with the study of Hering et al., in which the punicalagin metabolites ellagic acid and urolithin A strengthened or protected tight-junction-mediated epithelial function [19]. Ellagic acid increased resistance and reduced permeability in ileum-like Caco-2 monolayers, whereas urolithin A limited TNF-α-induced barrier loss and claudin dysregulation in colonic cells. The in vivo pattern of higher of ZO-1/occludin and reduction in endotoxemia extend this concept to polymicrobial sepsis. A relevant difference is that intact punicalagin produced little or no direct barrier benefit in some cell-culture conditions [19], whereas PUN was highly effective in our rats. This divergence is plausibly explained by gastric and intestinal hydrolysis to ellagic acid, microbiota-dependent urolithin formation, systemic immune and endothelial effects, and the inability of epithelial monolayers to reproduce the perfusion, microbial, and inflammatory complexity of CLP. Thus, the whole-animal response may reflect combined actions of PUN and its metabolites rather than a direct effect of the parent molecule alone.

Oxidative stress contributes to septic organ injury through lipid, protein, endothelial, and mitochondrial damage [12,13,14]. PPE and PUN were associated with lower MDA, TOS, and OSI and higher SOD, CAT, and TAS, indicating a coordinated redox response rather than reliance on a single assay. Nevertheless, antioxidant effects are context- and dose-dependent and do not automatically translate into therapeutic benefit, particularly for chemically complex preparations [29]. This caution is reinforced by the divergent pomegranate–sepsis literature [21,22,23,24,25,26].

The redox findings are consistent with Makled et al., who demonstrated that pomegranate reduced CLP-induced hepatic oxidative stress and inflammatory signaling through TLR4/NF-κB modulation [21], and with Ülker et al., who reported less severe visceral-organ injury following *P. granatum* peel powder treatment [24]. Our study extends these observations by showing that redox recovery occurred alongside preservation of the intestinal barrier, lower bacterial translocation, restoration of SIRT1/PGC-1α, and reduced injury signals from several organs. Differences in extract type and endpoints prevent direct quantitative comparison: Makled et al. emphasized hepatic molecular pathways, whereas Ülker et al. used peel powder and histopathological/microbiological outcomes. The broader response observed here may relate to the chemically enriched extract, the inclusion of two PPE doses and purified PUN, or the integrated analytical panel rather than necessarily indicating superior efficacy.

SIRT1 and PGC-1α coordinate metabolic adaptation and mitochondrial biogenesis, but protein concentrations are not functional mitochondrial measurements. Their higher abundance after PPE/PUN, together with lower lactate, is consistent with altered SIRT1/PGC-1α-associated signaling and metabolic adaptation. It does not prove restored oxidative phosphorylation, mitochondrial biogenesis, or improved mitochondrial function; lactate may also reflect perfusion, adrenergic drive, and substrate metabolism.

Supporting literature shows that punicalagin can activate protective autophagy and Akt/FoxO3a signaling in oxidative liver injury [30], while pomegranate polyphenols affect redox-sensitive metabolic and inflammatory pathways [21,31,32]. Our findings are similar in showing changes in protective signaling and reduced tissue injury, but differ in disease context and measured pathways. We did not quantify mitochondrial respiration, ATP, mitochondrial DNA copy number, respiratory complexes, or autophagic flux. Consequently, the SIRT1/PGC-1α response should be interpreted as an associative signaling finding, not direct proof that oxidative phosphorylation was restored. The reduction in lactate is concordant but cannot distinguish improved perfusion from intrinsic mitochondrial recovery.

Sepsis also reorganizes endocrine communication among adipose tissue, skeletal muscle, liver, endothelium, and immune cells. Extracellular NAMPT/visfatin and resistin may rise with inflammatory stress, whereas CTRP3, irisin, nesfatin-1, and myonectin participate in metabolic, vascular, and tissue-protective signaling [33,34,35,36,37,38]. In the present study, CLP increased visfatin/NAMPT and resistin and markedly decreased CTRP3, irisin, nesfatin-1, and myonectin. High-dose PPE and PUN shifted every mediator toward sham values. This coordinated pattern, together with lower endotoxin and lactate, supports partial normalization of immunometabolic homeostasis rather than an isolated change in one peptide.

The literature provides biological plausibility for the individual mediators but only limited direct comparison in polymicrobial sepsis. NAMPT is essential for intracellular NAD biosynthesis but can also function extracellularly as an inflammatory signal [33]; resistin links metabolic and inflammatory pathways [34]; CTRP3 can suppress hepatic glucose output [35]; irisin and myonectin connect skeletal muscle with systemic energy regulation [36,38]; and nesfatin-1 participates in appetite and neuroendocrine control [37]. Our results extend these largely non-septic observations by demonstrating simultaneous remodeling during CLP and reversal after PPE/PUN. The difference in experimental context is important: circulating concentrations cannot distinguish whether these mediators directly contributed to protection, reflected improved tissue function, or represented compensatory responses. Tissue expression, receptor activation, and blocking experiments would be required to establish causality.

The endothelial glycocalyx and microvascular endothelium form another critical interface between gut-derived signals and organ failure. Glycocalyx degradation releases syndecan-1, exposes adhesion molecules, increases permeability, and disrupts capillary flow [11]. Our CLP group showed large increases in syndecan-1 and E-selectin alongside endotoxemia, hyperlactatemia, and distant-organ injury. PPE 300 mg/kg reduced syndecan-1 by 69% and markedly lowered E-selectin; PUN generated a similar response. The fact that endothelial improvement paralleled preserved junctional proteins and reduced bacterial burden supports interruption of a gut–endothelial amplification loop.

Organ-specific findings reinforced this systemic interpretation. CLP increased KIM-1, creatinine, BUN, aminotransferases, cTnI, CK-MB, S100B, NSE, SP-D, and sRAGE, while decreasing BDNF. PPE and PUN reduced these abnormalities in a dose-responsive manner. Makled et al. similarly reported hepatic protection in CLP [21], and Ülker et al. observed reduced multi-organ histopathology with peel powder [24]. Our results are concordant but broader because renal tubular, cardiac, neurovascular, pulmonary epithelial, endothelial, and conventional biochemical indices were evaluated together. The explanation for this broad effect is likely multifactorial—lower endotoxin exposure, improved redox reserve, reduced glycocalyx injury, better microbial containment, and metabolic stabilization—rather than a direct action on every organ.

Some differences from previous work deserve attention. Ülker et al. reported no pancreatic lesions in their earlier peel-powder study [24], whereas moderate pancreatic injury occurred in our untreated CLP group and was reduced by PPE and PUN. Variation in ligation length, needle size and number of punctures, fecal extrusion, observation time, sex, supportive care, and lesion definitions can materially alter CLP severity and organ distribution [28]. In addition, our semi-quantitative grading captured proportional tissue involvement, whereas different sampling planes or scoring systems may miss focal lesions. Blinded digital morphometry and immunohistochemical confirmation would strengthen future comparisons.

The microbiological and survival findings are potentially important but require cautious interpretation. PPE and PUN groups had lower recoverable aerobic bacterial burdens in peritoneal lavage, spleen, and lung, and higher observed 48 h survival than untreated CLP. These findings are directionally similar to reports of prolonged survival with *P. granatum* peel extract, beneficial interaction between PUN and meropenem, and reduced culture positivity after peel-powder treatment [22,23,24]. Conversely, other studies reported increased peritoneal burden or adverse immune effects under different pomegranate exposure conditions [25,26]. The present aerobic culture approach did not characterize strict anaerobes, the full polymicrobial community, susceptibility, or microbiome composition; counts below the quantification limit do not establish sterilization or a mechanism of bacterial clearance.

In contrast, Tavasoli et al. reported increased peritoneal bacterial load and mortality after pomegranate extract exposure [25], and prolonged high-dose supplementation suppressed neutrophil myeloperoxidase-related responses [26]. These results differ directly from our reduced dissemination and improved survival. Several factors may explain the divergence: pretreatment versus post-CLP treatment, duration of exposure, extract composition, administered dose, CLP severity, sex, microbiota, and the balance between antioxidant/anti-inflammatory action and early neutrophil-dependent pathogen clearance. Prolonged high-dose pretreatment may dampen antimicrobial effector function before infection is established, whereas a single post-insult dose may reduce barrier and oxidative injury without comparable immune preconditioning. The discrepancy demonstrates that a botanical preparation cannot be assumed beneficial solely because it has antioxidant activity. Our below-detection cultures also do not establish sterilization; isolate-specific MIC/MBC, time-kill, tissue exposure, and antibiotic-combination studies remain necessary.

Clinical scores, stool consistency, and grimace observations changed in the same direction as biochemical outcomes. These ordinal observations offer functional context but remain subjective. The available record does not document blinded clinical scoring; observer bias therefore cannot be excluded and is acknowledged as a limitation. Clinical improvement in this animal model should not be equated with clinical therapeutic efficacy.

PUN 50 mg/kg generally matched or modestly exceeded PPE 300 mg/kg, but the comparison is not exposure-matched. PPE 300 mg/kg theoretically contained approximately 105 mg/kg total punicalagin based on the measured 34.9% composition, before accounting for recovery, stability, absorption, metabolism, and matrix effects. Because the PPE and PUN groups were neither equimolar nor pharmacokinetically matched, the experiment cannot determine whether punicalagin quantitatively accounts for PPE activity. It demonstrates only that purified PUN at the tested dose produced a similar phenotype. Plasma and tissue concentrations of punicalagin, ellagic acid, and urolithins were not measured, so the biologically active exposure remains unknown.

NLRP3/pyroptosis remains a future hypothesis. Although punicalagin has affected TXNIP/NLRP3-associated pyroptosis in another disease model [39], NLRP3, ASC, caspase-1, gasdermin-D, mature IL-1β/IL-18, and pyroptotic cell death were not measured here. These pathways therefore cannot explain the current results and are not presented as demonstrated mechanisms.

The strongest multidomain responses occurred with PPE 300 mg/kg and PUN 50 mg/kg, but these regimens are reference doses only for confirmatory rodent experiments. The study did not include standard antibiotics, pharmacokinetics, direct permeability, or both sexes; it cannot support human dosing or clinical readiness. Future experiments should use exposure-matched PPE/PUN comparisons and test each intervention alone and with standard antimicrobial therapy [7,23].

Strengths include the polymicrobial CLP model, two PPE doses, a purified-PUN comparator, a PPE-per-se group, and integration of multiple outcome domains. Major limitations are *n* = 8/group; imprecise survival proportions (deaths: CLP 6/8, PPE 150 3/8, PPE 300 1/8, PUN 0/8, Sham 0/8, PPE per se 0/8); absence of a standard-antibiotic arm; female-only animals; unblinded or undocumented blinding for clinical scores; semi-quantitative histology with incomplete section/field documentation; aerobic culture without comprehensive anaerobic/species characterization; no direct permeability, cytokine, mitochondrial-function, pharmacokinetic, or pathway-intervention assays; lack of biomarker-family multiplicity control; and botanical provenance not specified in the submitted materials. Terminal samples were collected at humane endpoints or at 48 h, creating substantial time-related confounding. These limitations restrict causal, mechanistic, and translational inference.

Future studies should prespecify primary outcomes, retain individual-level data, report effect sizes with 95% confidence intervals, control false discovery within endpoint families, and use synchronized serial sampling or separate cohorts for survival and fixed-time biomarkers. Both sexes, standard antibiotics, exposure-matched PPE/PUN groups, complete botanical voucher documentation, validated LC-MS batch characterization, direct permeability assays, mitochondrial functional testing, cytokine panels, anaerobic/species-resolved microbiology, pharmacokinetics, and blinded quantitative histomorphometry are required before translational claims can be advanced.

## 5. Conclusions

In this 48 h CLP experiment, PPE and punicalagin were associated with improved intestinal barrier markers, lower circulating permeability and injury indices, reduced bacterial dissemination, and less severe histopathological changes relative to untreated CLP animals. PPE 300 mg/kg and punicalagin 50 mg/kg also showed higher observed 48 h survival proportions. These findings indicate associations within this exploratory animal study and do not establish clinical efficacy, mechanistic mediation, or a graded dose–response relationship.

The results should be interpreted in light of the small group size, female-only design, single 48 h observation window, non-equimolar treatment comparison, limited analytical provenance, and representative rather than fully quantified figure panels. Confirmation requires independent replication with prespecified outcomes, verified exposure, complete botanical and analytical records, and histopathology based on documented section and field sampling. No human therapeutic or safety conclusion can be drawn from the present data.

## Figures and Tables

**Table 1:** Intestinal barrier integrity, mucosal permeability, and systemic endotoxemia across experimental groups.

Group (mg/kg; *n* = 8)	Ileum ZO-1 (µg/mg Protein)	Ileum Occludin (µg/mg Protein)	Serum DAO (U/L)	Serum D-Lactate (µg/mL)	Plasma Endotoxin (EU/mL)
Sham	14.2 ± 1.1 ^a^	11.8 ± 0.9 ^a^	18.4 ± 1.6 ^d^	4.2 ± 0.5 ^d^	0.08 ± 0.01 ^d^
CLP	3.6 ± 0.4 ^d^	2.8 ± 0.3 ^d^	86.5 ± 7.2 ^a^	28.6 ± 2.4 ^a^	0.94 ± 0.08 ^a^
CLP + PPE 150	7.8 ± 0.7 ^c^	6.2 ± 0.6 ^c^	52.1 ± 4.8 ^b^	16.4 ± 1.5 ^b^	0.48 ± 0.04 ^b^
CLP + PPE 300	11.5 ± 1.0 ^b^	9.4 ± 0.8 ^b^	31.2 ± 2.9 ^c^	8.7 ± 0.8 ^c^	0.21 ± 0.02 ^c^
CLP + PUN 50	12.1 ± 0.9 ^b^	10.1 ± 0.9 ^ab^	28.6 ± 2.5 ^c^	7.9 ± 0.7 ^c^	0.18 ± 0.02 ^c^
PPE per se 300	14.5 ± 1.2 ^a^	12.0 ± 1.0 ^a^	17.9 ± 1.5 ^d^	4.0 ± 0.4 ^d^	0.07 ± 0.01 ^d^
*p*-value	<0.001	<0.001	<0.001	<0.001	<0.001

Values are expressed as mean ± standard deviation (SD) for *n* = 8 animals per group. Different lowercase superscript letters (a–d) within a column designate statistically significant differences between groups (*p* < 0.05, one-way ANOVA followed by Tukey’s post hoc test). CLP, cecal ligation and puncture; PPE, pomegranate peel extract; PUN, punicalagin; ZO-1, zonula occludens-1; DAO, diamine oxidase; EU, endotoxin units.

**Table 2:** Circulating redox balance indices and sentinel neurovascular and pulmonary injury markers.

Group (mg/kg; *n* = 8)	MDA (nmol/mL)	SOD (U/mL)	CAT (kU/L)	TAS (mmol Trolox Eq/L)	TOS (µmol H_2_O_2_ Eq/L)	OSI (AU)	S100B (pg/mL)	SP-D (ng/mL)
Sham	1.82 ± 0.16 ^d^	22.4 ± 1.9 ^a^	28.6 ± 2.4 ^a^	2.14 ± 0.18 ^a^	7.1 ± 0.6 ^d^	0.33 ± 0.03 ^d^	42.5 ± 3.8 ^d^	9.8 ± 0.9 ^d^
CLP	7.45 ± 0.62 ^a^	9.1 ± 0.8 ^d^	11.4 ± 1.0 ^d^	0.72 ± 0.06 ^d^	29.8 ± 2.5 ^a^	4.14 ± 0.36 ^a^	268.4 ± 24.1 ^a^	54.2 ± 4.8 ^a^
CLP + PPE 150	4.60 ± 0.38 ^b^	13.8 ± 1.2 ^c^	17.5 ± 1.5 ^c^	1.18 ± 0.10 ^c^	19.4 ± 1.7 ^b^	1.64 ± 0.15 ^b^	168.5 ± 15.2 ^b^	34.6 ± 3.1 ^b^
CLP + PPE 300	2.65 ± 0.22 ^c^	18.2 ± 1.5 ^b^	23.1 ± 2.0 ^b^	1.75 ± 0.15 ^b^	11.8 ± 1.0 ^c^	0.67 ± 0.06 ^c^	78.2 ± 7.1 ^c^	17.4 ± 1.5 ^c^
CLP + PUN 50	2.38 ± 0.20 ^c^	19.6 ± 1.7 ^b^	24.8 ± 2.2 ^b^	1.88 ± 0.16 ^b^	10.2 ± 0.9 ^c^	0.54 ± 0.05 ^c^	69.4 ± 6.2 ^c^	15.1 ± 1.3 ^c^
PPE per se 300	1.75 ± 0.14 ^d^	23.1 ± 2.0 ^a^	29.4 ± 2.6 ^a^	2.20 ± 0.19 ^a^	6.8 ± 0.5 ^d^	0.31 ± 0.03 ^d^	40.8 ± 3.5 ^d^	9.2 ± 0.8 ^d^
*p*-value	<0.001	<0.001	<0.001	<0.001	<0.001	<0.001	<0.001	<0.001

Values are mean ± SD (*n* = 8 per group). Distinct superscript letters (a–d) within each column indicate statistically significant differences (*p* < 0.05, one-way ANOVA followed by Tukey’s post hoc test). MDA, malondialdehyde; SOD, superoxide dismutase; CAT, catalase; TAS, total antioxidant status; TOS, total oxidant status; OSI, oxidative stress index; S100B, S100 calcium-binding protein B; SP-D, surfactant protein-D.

**Table 3:** Hepatic mitochondrial biogenesis regulators, myokine signaling, endothelial glycocalyx shedding, and renal tubular injury across experimental groups.

Group (mg/kg; *n* = 8)	Hepatic SIRT1 (ng/mg Protein)	Hepatic PGC-1α (ng/mg Protein)	Myonectin (ng/mL)	Syndecan-1 (ng/mL)	KIM-1 (pg/mL)
Sham	8.4 ± 0.7 ^a^	6.2 ± 0.5 ^a^	48.5 ± 4.2 ^a^	2.1 ± 0.2 ^d^	112.4 ± 10.5 ^d^
CLP	2.1 ± 0.2 ^d^	1.4 ± 0.1 ^d^	14.2 ± 1.3 ^d^	16.8 ± 1.5 ^a^	845.6 ± 72.4 ^a^
CLP + PPE 150	4.5 ± 0.4 ^c^	3.1 ± 0.3 ^c^	26.8 ± 2.4 ^c^	10.4 ± 0.9 ^b^	495.2 ± 41.8 ^b^
CLP + PPE 300	6.8 ± 0.6 ^b^	4.9 ± 0.4 ^b^	38.9 ± 3.5 ^b^	5.2 ± 0.5 ^c^	265.4 ± 22.6 ^c^
CLP + PUN 50	7.2 ± 0.6 ^b^	5.2 ± 0.5 ^b^	41.2 ± 3.8 ^ab^	4.6 ± 0.4 ^c^	238.1 ± 20.5 ^c^
PPE per se 300	8.6 ± 0.8 ^a^	6.4 ± 0.6 ^a^	49.2 ± 4.5 ^a^	2.0 ± 0.2 ^d^	108.6 ± 9.8 ^d^
*p*-value	<0.001	<0.001	<0.001	<0.001	<0.001

Values are mean ± SD (*n* = 8 per group). Superscripts (a–d) within columns indicate significant inter-group differences (*p* < 0.05, one-way ANOVA/Tukey). SIRT1, sirtuin-1; PGC-1α, peroxisome proliferator-activated receptor-γ coactivator-1α; KIM-1, kidney injury molecule-1.

**Table 4:** Circulating immunometabolic adipokine and myokine profiles across experimental groups.

Group (mg/kg; *n* = 8)	Visfatin/NAMPT (ng/mL)	Resistin (ng/mL)	CTRP3 (ng/mL)	Irisin (ng/mL)	Nesfatin-1 (ng/mL)
Sham	6.2 ± 0.6 ^d^	5.1 ± 0.5 ^d^	142.5 ± 12.4 ^a^	8.6 ± 0.8 ^a^	3.12 ± 0.28 ^a^
CLP	28.4 ± 2.6 ^a^	24.8 ± 2.2 ^a^	38.6 ± 3.5 ^d^	2.1 ± 0.2 ^d^	0.85 ± 0.08 ^d^
CLP + PPE 150	17.5 ± 1.6 ^b^	15.2 ± 1.4 ^b^	74.8 ± 6.9 ^c^	4.5 ± 0.4 ^c^	1.64 ± 0.15 ^c^
CLP + PPE 300	9.8 ± 0.9 ^c^	8.4 ± 0.8 ^c^	118.2 ± 10.5 ^b^	6.9 ± 0.6 ^b^	2.45 ± 0.22 ^b^
CLP + PUN 50	8.9 ± 0.8 ^c^	7.6 ± 0.7 ^c^	124.6 ± 11.2 ^b^	7.3 ± 0.7 ^b^	2.60 ± 0.24 ^b^
PPE per se 300	6.0 ± 0.5 ^d^	4.9 ± 0.4 ^d^	145.1 ± 13.0 ^a^	8.8 ± 0.8 ^a^	3.18 ± 0.29 ^a^
*p*-value	<0.001	<0.001	<0.001	<0.001	<0.001

Values are mean ± SD (*n* = 8 per group). Distinct superscript letters (a–d) within columns indicate significant differences (*p* < 0.05, one-way ANOVA/Tukey). NAMPT, nicotinamide phosphoribosyltransferase; CTRP3, C1q/TNF-related protein 3.

**Table 5:** Serum biochemical markers of hepatic, renal, cardiac, and metabolic injury across experimental groups.

Group (mg/kg; *n* = 8)	AST (U/L)	ALT (U/L)	Creatinine (mg/dL)	BUN (mg/dL)	cTnI (ng/mL)	CK-MB (U/L)	Glucose (mg/dL)	Lactate (mmol/L)
Sham	72.5 ± 6.1 ^d^	34.8 ± 3.1 ^d^	0.38 ± 0.03 ^d^	15.4 ± 1.3 ^d^	0.03 ± 0.01 ^d^	135.2 ± 11.8 ^d^	118.4 ± 9.2 ^a^	1.35 ± 0.12 ^d^
CLP	312.4 ± 26.8 ^a^	162.5 ± 14.2 ^a^	1.85 ± 0.16 ^a^	71.2 ± 6.4 ^a^	0.74 ± 0.07 ^a^	524.6 ± 42.5 ^a^	54.8 ± 4.9 ^d^	6.42 ± 0.58 ^a^
CLP + PPE 150	188.6 ± 16.2 ^b^	96.4 ± 8.5 ^b^	1.18 ± 0.11 ^b^	46.8 ± 4.1 ^b^	0.38 ± 0.04 ^b^	338.5 ± 28.4 ^b^	79.2 ± 7.1 ^c^	3.95 ± 0.36 ^b^
CLP + PPE 300	108.4 ± 9.6 ^c^	48.2 ± 4.3 ^c^	0.58 ± 0.05 ^c^	24.6 ± 2.2 ^c^	0.11 ± 0.01 ^c^	192.4 ± 16.5 ^c^	104.5 ± 8.8 ^b^	2.05 ± 0.18 ^c^
CLP + PUN 50	98.2 ± 8.7 ^c^	44.1 ± 3.9 ^c^	0.52 ± 0.04 ^c^	22.1 ± 1.9 ^c^	0.09 ± 0.01 ^c^	178.6 ± 15.2 ^c^	109.8 ± 9.4 ^ab^	1.88 ± 0.16 ^c^
PPE per se 300	70.8 ± 5.9 ^d^	33.5 ± 2.9 ^d^	0.37 ± 0.03 ^d^	14.8 ± 1.2 ^d^	0.03 ± 0.01 ^d^	131.0 ± 10.9 ^d^	120.2 ± 9.8 ^a^	1.30 ± 0.11 ^d^
*p*-value	<0.001	<0.001	<0.001	<0.001	<0.001	<0.001	<0.001	<0.001

Values are mean ± SD (*n* = 8 per group). Distinct superscript letters (a–d) within columns denote significant differences (*p* < 0.05, one-way ANOVA/Tukey). AST, aspartate aminotransferase; ALT, alanine aminotransferase; BUN, blood urea nitrogen; cTnI, cardiac troponin I; CK-MB, creatine kinase-myocardial band.

**Table 6:** Circulating neurovascular, pulmonary epithelial, and endothelial activation markers across experimental groups.

Group (mg/kg; *n* = 8)	S100B (pg/mL)	NSE (ng/mL)	BDNF (pg/mL)	SP-D (ng/mL)	sRAGE (pg/mL)	E-Selectin (ng/mL)
Sham	42.5 ± 3.8 ^d^	3.85 ± 0.32 ^d^	328.6 ± 26.4 ^a^	9.8 ± 0.9 ^d^	76.4 ± 6.8 ^d^	2.85 ± 0.24 ^d^
CLP	268.4 ± 24.1 ^a^	21.40 ± 1.95 ^a^	98.4 ± 9.2 ^d^	54.2 ± 4.8 ^a^	382.5 ± 32.4 ^a^	16.95 ± 1.45 ^a^
CLP + PPE 150	168.5 ± 15.2 ^b^	13.25 ± 1.20 ^b^	182.4 ± 16.5 ^c^	34.6 ± 3.1 ^b^	245.8 ± 21.6 ^b^	10.80 ± 0.95 ^b^
CLP + PPE 300	78.2 ± 7.1 ^c^	5.80 ± 0.52 ^c^	284.5 ± 24.1 ^b^	17.4 ± 1.5 ^c^	120.5 ± 11.2 ^c^	4.90 ± 0.42 ^c^
CLP + PUN 50	69.4 ± 6.2 ^c^	5.15 ± 0.46 ^c^	296.8 ± 25.8 ^ab^	15.1 ± 1.3 ^c^	110.2 ± 9.8 ^c^	4.25 ± 0.38 ^c^
PPE per se 300	40.8 ± 3.5 ^d^	3.70 ± 0.30 ^d^	335.2 ± 28.1 ^a^	9.2 ± 0.8 ^d^	72.8 ± 6.2 ^d^	2.70 ± 0.22 ^d^
*p*-value	<0.001	<0.001	<0.001	<0.001	<0.001	<0.001

Values are mean ± SD (*n* = 8 per group). Distinct superscript letters (a–d) within columns indicate significant differences (*p* < 0.05, one-way ANOVA/Tukey). S100B, S100 calcium-binding protein B; NSE, neuron-specific enolase; BDNF, brain-derived neurotrophic factor; SP-D, surfactant protein-D; sRAGE, soluble receptor for advanced glycation end-products.

**Table 7:** Quantitative bacterial burden across anatomical compartments.

Group (mg/kg; *n* = 8)	Peritoneal Lavage (log10 CFU/mL)	Spleen Homogenate (log10 CFU/g)	Lung Homogenate (log10 CFU/g)
Sham	<1.00	<1.00	<1.00
CLP	5.84 ± 0.42 ^a^	4.62 ± 0.38 ^a^	3.95 ± 0.31 ^a^
CLP + PPE 150	3.72 ± 0.31 ^b^	2.84 ± 0.25 ^b^	2.15 ± 0.20 ^b^
CLP + PPE 300	1.85 ± 0.16 ^c^	1.20 ± 0.12 ^c^	<1.00 ^c^
CLP + PUN 50	1.64 ± 0.14 ^c^	<1.00 ^c^	<1.00 ^c^
PPE per se 300	<1.00	<1.00	<1.00
*p*-value	<0.001	<0.001	<0.001

Quantitative culture values are expressed as mean ± SD (log10 CFU/unit) for *n* = 8 animals per group. Distinct superscript letters (a–c) denote significant differences (*p* < 0.05, one-way ANOVA followed by Tukey’s post hoc test). Bacterial counts < 1.00 log10 CFU represent values below the analytical quantification limit. CFU, colony-forming units; CLP, cecal ligation and puncture; PPE, pomegranate peel extract; PUN, punicalagin.

**Table 8:** Clinical status, behavioral impairment, rat grimace pain score, stool consistency, and 48 h survival dynamics across experimental cohorts.

Experimental Group (mg/kg; *n* = 8)	Behavioral Score (0–5)	Clinical Sepsis Score (0–4)	Rat Grimace Pain Score (0–3)	Stool Consistency	Surviving Animals (n/N)	48 h Cumulative Survival (%)
Sham	0 (Normal) ^d^	0.0 ± 0.0 ^d^	0 (None) ^d^	Normal/Formed	8/8	100
CLP	4 (Severe lethargy/Prostration) ^a^	3.8 ± 0.4 ^a^	3 (Severe distortion) ^a^	Diarrheic/Watery	2/8	25.0
CLP + PPE 150	2 (Mild lethargy) ^b^	2.1 ± 0.3 ^b^	2 (Moderate grimace) ^b^	Soft/Unformed	5/8	62.5
CLP + PPE 300	1 (Active/Minor impairment) ^c^	0.8 ± 0.2 ^c^	1 (Mild grimace) ^c^	Normal/Formed	7/8	87.5
CLP + PUN 50	1 (Active/Alert) ^c^	0.6 ± 0.2 ^c^	0–1 (Minimal/Absent) ^cd^	Normal/Formed	8/8	100
PPE per se 300	0 (Normal) ^d^	0.0 ± 0.0 ^d^	0 (None) ^d^	Normal/Formed	8/8	100
*p*-value	<0.001	<0.001	<0.001	—	—	<0.01

Values are expressed as mean ± SD for continuous scores (*n* = 8 per group) and median/interquartile descriptions for ordinal scores. Continuous parametric data (Clinical Sepsis Score) were analyzed using one-way ANOVA followed by Tukey’s post hoc test. Ordinal behavioral and grimace pain scores were analyzed using the non-parametric Kruskal–Wallis test followed by Dunn’s post hoc test for multiple comparisons. Distinct lowercase superscript letters (a–d) within a column indicate statistically significant differences between groups (*p* < 0.05). Cumulative 48 h survival curves were analyzed using the Kaplan–Meier method and log-rank (Mantel–Cox) test. CLP, cecal ligation and puncture; PPE, pomegranate peel extract; PUN, punicalagin. Behavioral score: 0 = normal active, 1 = slightly decreased movement, 2 = mild lethargy, 3 = moderate hypoactivity, 4 = severe lethargy/prostration, 5 = moribund.

**Table 9:** Semi-quantitative histopathological injury grading across target organs.

Group (mg/kg; *n* = 8)	Liver Degeneration/Necrosis	Lung Infiltration/Hyperemia/Septal Thickening	Cardiac Degeneration/Infiltration	Renal Tubular Degeneration	Splenic Follicular Hyperplasia	Pancreatic Degeneration/Inflammation
Sham	−	−	−	−	−	−
CLP	+++	+++	++	+++	+++	++
CLP + PPE 150	++	++	+	++	++	+
CLP + PPE 300	+	+	−/+	+	+	−/+
CLP + PUN 50	−/+	+	−/+	+	+	−
PPE per se 300	−	−	−	−	−	−

Semi-quantitative histopathological injury grades: (−), absent/minimal structural changes (<10% of tissue section); (+), mild damage (10–30% of tissue section); (++), moderate tissue disruption (30–50% of tissue section); (+++), severe diffuse necrosis and architectural breakdown (>50% of tissue section). Values represent descriptive composite histopathological grades across experimental cohorts; inferential *p*-values cannot be derived from group-level grades alone. Animal-level ordinal scores are needed for a valid group comparison.

## Data Availability

The data supporting the findings of this study are available from the corresponding author upon reasonable request.
